# Comparison of Combined Dissipation Behaviors and Dietary Risk Assessments of Thiamethoxam, Bifenthrin, Dinotefuran, and Their Mixtures in Tea

**DOI:** 10.3390/foods13193113

**Published:** 2024-09-29

**Authors:** Tiancai Wang, Yongzhong Qian, Jieqiong Wang, Xueyan Yin, Qifu Liang, Guangqin Liao, Xiabing Li, Jing Qiu, Yanyang Xu

**Affiliations:** 1 Key Laboratory of Agro-Product Quality and Safety, Institute of Quality Standards and Testing Technology for Agro-Products, Chinese Academy of Agricultural Sciences, Beijing 100081, China; 18810583803@163.com (T.W.); qianyongzhong@caas.cn (Y.Q.); lgqlightcheng@163.com (G.L.); lixiabing123452023@163.com (X.L.); qiujing@caas.cn (J.Q.); 2 Hubei Key Laboratory of Nutritional Quality and Safety of Agro-Products, Laboratory of Quality & Safety Risk Assessment for Agro-Products (Wuhan), Institute of Quality Standard and Testing Technology for Agro-Products, Hubei Academy of Agricultural Sciences, Ministry of Agriculture and Rural Affairs, Wuhan 430064, China; 3 Changzhou Supervision and Inspection Center for Quality of Agricultural, Livestock and Aquatic Products, Changzhou 213001, China; yuanhao614@126.com (J.W.); yh614@163.com (X.Y.); 4 Fujian Key Laboratory of Agro-Products Quality & Safety, Institute of Quality Standards & Testing Technology for Agro-Products, Fujian Academy of Agricultural Sciences, Fuzhou 350003, China; k861225@163.com

**Keywords:** tea, joint application, half-life, risk assessment

## Abstract

In the tea-planting process, insecticides are commonly combined, potentially prolonging the pre-harvest interval and heightening the risk of dietary exposure. This study focused on three frequently used insecticides in tea cultivation: thiamethoxam, bifenthrin, and dinotefuran, aiming to investigate their dissipation behaviors and associated dietary risks upon individual and simultaneous application. The dissipation kinetics of thiamethoxam, bifenthrin, and dinotefuran were successfully characterized by first-order kinetics, yielding respective half-lives of 5.44, 9.81, and 10.16 days. Upon joint application, the dissipation half-lives of thiamethoxam and bifenthrin were notably prolonged compared with their individual applications, resulting in final concentrations after 28 days that were correspondingly elevated by 1.41 and 1.29 times. Assessment of the dietary intake risk revealed that the chronic and acute risk quotients associated with thiamethoxam and bifenthrin escalated by 1.44–1.59 times following their combined application. Although dietary risks associated with Tianmuhu white tea, as determined by the exposure assessment model, were deemed acceptable, the cumulative risks stemming from pesticide mixtures across various dietary sources warrant attention. Molecular docking analyses further unveiled that thiamethoxam and bifenthrin competitively bound to glutathione S-transferase (GST) at amino acid residues, notably at the 76th GLU and the 25th PHE, pivotal in the metabolism and absorption of exogenous substances. Moreover, the interactions between P-glycoprotein and pesticides during transport and absorption were likely to influence dissipation behaviors post-joint application. This research offers valuable insights and data support for optimizing joint pesticide application strategies and assessing risks associated with typical pesticides used in tea cultivation.

## 1. Introduction

Tea (*Camellia sinensis*) has emerged as a globally beloved beverage [1]. However, tea cultivation faces significant challenges from various pests and diseases. Among them, the tea green leafhopper (*Empoasca onukii Matsuda*), tea geometrid (*Ectropis oblique hypulina Wehrli*), and stick tea thrip (*Dendrothrips minowai Priesner*) are notable, inflicting substantial damage on tea yield and quality [2]. To combat these threats, farmers often opt for pesticide mixtures containing two or more varieties, as this method is considered more effective than individual pesticide applications [3,4]. For example, emamectin benzoate and thiamethoxam were used in combination to control both tea geometrid and tea green leafhopper [5]. By offering a broader spectrum of pest control, mixed pesticides enhance efficacy in tea cultivation [6]. Pesticides play a crucial role in managing pests, pathogens, and weeds in tea cultivation. Despite undergoing natural processes like degradation through hydrolysis, photolysis, and microbial breakdown in tea gardens, leading to partial degradation, residues of pesticides often persist in tea [7]. Consequently, concerns about the potential health effects of pesticide exposure have arisen among tea consumers. In China, neonicotinoids and pyrethroids stand out as the most commonly used insecticides, either individually or in mixtures, to control pests in tea plantations [8]. Prominent examples include thiamethoxam, bifenthrin, and dinofuran [9].

Monitoring of neonicotinoid pesticide residues in 220 samples of Chinese tea revealed that 90.5% of the samples contained at least one neonicotinoid insecticide, with thiamethoxam and dinotefuran being the most prominent [10]. Thiamethoxam, acting as an acetylcholinesterase receptor inhibitor, disrupts the central nervous systems of insects, leading to loss of function [11]. Dinotefuran, a third-generation neonicotinoid insecticide, features a tetrahydrofuran group replacing the chloropyridinyl and chlorothiazolyl groups, endowing it with superior systemic action and significant insecticidal activity at low doses [12]. In the tea-planting process, simultaneous use of multiple pyrethroid insecticides has been observed, as reported by Zhuang through monitoring residue levels in tea leaves from the Beijing market. Among these, bifenthrin showed the highest residue level, reaching 491.24 μg/kg [13]. Bifenthrin functions by increasing sodium ion permeability in the nervous system, leading to prolonged activation of sodium ion channels and consequent overexcitation, ultimately resulting in death [14].

However, simultaneous application of pesticides at close intervals can result in the presence of multiple pesticide residues [15]. Lu conducted an examination of pesticide residue levels in 1629 tea leaf samples, revealing that 20 pesticides were detected in 3 samples, and more than 10 pesticides were detected in 40 samples, indicating potential simultaneous exposure of tea consumers to multiple pesticides [16]. Prolonged exposure to multiple pesticides can disrupt the endocrine system, nervous system, reproductive system, and other chronic toxicities [17]. Furthermore, extended pre-harvest intervals also may increase risks to food safety [18]. Therefore, investigating the residue behaviors and dietary risks associated with the joint application of insecticides in tea leaves is of paramount importance.

In this study, the dissipation behaviors and dietary risks associated with thiamethoxam, bifenthrin, and dinotefuran in tea under co-application conditions were analyzed. Tianmuhu white tea was selected as the research subject, and the dynamic dissipation behaviors of single and multiple insecticides during the tea-planting process were investigated using gas chromatogram–mass spectrometry (GC-MS) and liquid chromatography–tandem mass spectrometry (LC-MS/MS) methods. Risk assessment of dietary intake provided chronic and acute risk quotients of insecticides. Molecular docking techniques were employed to explore the interaction of pesticide molecules with metabolizing enzymes in tea metabolism, thereby elucidating the molecular mechanisms underlying changes in dissipation behavior following joint application. This research would provide guidance and data support for joint application and risk evaluation of typical pesticides in tea.

## 2. Materials and Methods

### 2.1. Materials and Chemicals

Thiamethoxam, bifenthrin, and dinotefuran standard solutions (1000 mg/L) were obtained from Nanjing Dun Ken Scientific Equipment Co., Ltd. (Nanjing, China). Water dispersible granules (WDGs) containing 25% thiamethoxam were purchased from Shandong Yi Jia Agricultural Chemical Co., Ltd. (Weifang, China), with a recommended dose of 24.24–36.36 g/acre. An emulsion in water (EW) containing 20% bifenthrin was acquired from Shanghai Yue Lian Chemical Co., Ltd. (Shanghai, China), with a recommended dose of 72.73–90.91 mL/acre. A microemulsion (ME) containing 10% dinotefuran was purchased from Hebei Shan Si Biotechnology Co., Ltd. (Shijiazhuang, China), with a recommended dose of 363.64–484.85 mL/acre.

Acetone and acetonitrile (chromatographic grade) were purchased from CNW Company (Duesseldorf, Germany). An extraction salt package (composed of 5.5 g MgSO_4_, 1.5 g NaCl, and 1 g of sodium citrate alongside 1.5 g of disodium citrate buffer system) and a purification kit (containing 100 mg PSA, 100 mg C18, 100 mg GCB, and 600 mg anhydrous MgSO_4_) were purchased from Beijing Ben Li Technology Co., Ltd. (Beijing, China). Ultrapure water was obtained from a Milli-Q ultrapure water apparatus (Darmstadt, Germany).

### 2.2. Field Trials

Field trials were performed in Changzhou, China, in 2022, aligned with the guidelines for testing of pesticide residues in crops [19]. The area of each experimental plot was 30 square meters, in which no experimental pesticides had been used. Dissipation kinetics and terminal residues of three insecticides were investigated. Based on the pesticide product labels, tea plants were sprayed with 0.27 g thiamethoxam commercial product, and/or 0.67 mL bifenthrin formulation, and/or 3.60 mL dinotefuran formulation across the experimental plots per 30 square meters. The pre-harvest intervals of thiamethoxam, bifenthrin, and dinotefuran on tea trees occurred at 3, 7, and 7 days, respectively. Simultaneously, an identically sized experimental plot with no pesticide application was performed to serve as the control group. A sample of tea measuring 0.3 kg was randomly harvested from experimental plots 0.08, 1, 3, 5, 7, 14, 21, 28, 35, and 60 days after spraying. Two parallel samples were utilized for each group. The weather conditions on the day of sampling were recorded (Table S1). Samples were homogenized and maintained at −20 °C until analysis.

### 2.3. Sample Preparation

Tea samples (2.0 g) were placed into an extraction tube with 4 mL water and 10 mL acetonitrile. The extraction and purification were performed using a solid-phase dispersed extraction sample processing machine (Sio-dSPE), drawing on procedures from a previous study [20]. Specifically, the procedure was conducted as follows. Firstly, the sample was oscillated at 1000 r/min for 10 min. Next, it underwent centrifugation at a speed of 4000 r/min for 5 min. Following this, the sample was oscillated again at 1000 r/min for 3 min. Finally, it was centrifuged once more at 4000 r/min for 3 min. After the treatment, the supernatant was filtered through a 0.22 μm microporous filter membrane and used for mass spectrometry analysis [21].

### 2.4. GC-MS Analysis

Bifenthrin was characterized by in-line gel permeation chromatography (GPC)–gas chromatography–triple quadrupole mass spectrometry (GC-MS-TQ8030, Shimadzu Corporation, Kyoto, Japan). The purification utilizing GPC was conducted with a CLNpak EV-200 chromatographic column (150 mm × 2 mm, Shimadzu Corporation, Kyoto, Japan). The mobile phase was composed of acetone and cyclohexane (3:7). The flow rate was 0.1 mL/min, and the column temperature was maintained at 40 °C. The injection volume was 10 µL. The chromatographic separation was carried out with a DB-5MS column (25 m × 0.25 mm, 0.25 μm, Agilent Corporation, CA, USA). The capillary column temperature was set at 82 °C for the initial 5 min and then increased at 8 °C/min to 300 °C, where it was held for 7.75 min. The total analysis time was 40 min. Mass spectrometry was conducted in electron impact (EI) mode, with an electron energy of 70 eV and a solvent delay time of 9 min. The collision gas was nitrogen (purity of at least 99.999%). MS data were obtained in multiple reaction monitoring (MRM) mode.

### 2.5. LC-MS/MS Analysis

Thiamethoxam and dinotefuran were characterized via liquid chromatography–tandem mass spectrometry (8060 LC-MS/MS, SHIMADZU Corporation, Kyoto, Japan) using a Hypersil GOLD (100 mm × 2.1 mm, 3 μm) column. The mobile phase A was composed of 5 mM ammonium acetate with 0.02% formic acid, and mobile phase B was methanol. The flow rate was 0.4 mL/min. The gradient elution procedure was 1 min, 10% B; 2 min, 50% B; 7 min, 95% B; and 10 min, 10% B. The total analysis time was 10 min. The interface, DL, and heat block temperatures were precisely maintained at 300 °C, 250 °C, and 400 °C, respectively.

### 2.6. Method Performances

The analytical methods were validated following the SANTE guidance document [22] and NY/T 788–2018, using linearity, the limit of detection (LOD), the limit of quantification (LOQ), accuracy, and precision. To eliminate the matrix effect, linearity was estimated by the linear correlation coefficient of the matrix-matched calibration curves equation in tea matrix extracts. The LODs and LOQs for each insecticide and metabolite were calculated by the minimum concentration with a signal-to-noise (S/N) ratio of 3 and 10, respectively. The accuracy and precision of the method were assessed via recovery experiments with two spiked levels (10 and 100 μg/L) across six replicates.

### 2.7. Dissipation Kinetics of Insecticides

The first-order kinetics equation was employed to investigate the dissipation kinetics of thiamethoxam, bifenthrin, and dinotefuran in tea samples. The half-life (t_1/2_, day) was obtained using the following equations:

C_t_ = C_0_e^−kt^ (1)

t_1/2_ = ln(2)/k (2)

where C_t_ and C_0_ indicate the pesticide residue concentrations (mg/kg) at time t and time 0 (day), respectively, while k is the dissipation rate.

### 2.8. Molecular Docking

Molecular docking was employed to examine the mechanism of combined dissipation between active ingredients and key targets. The structures of small molecules, thiamethoxam, bifenthrin, and dinotefuran were obtained from the PubChem database for docking. Proteins with UniProt IDs A0A7J7GM45 (glutathione S-transferase, GST), A0A7J7GXR7 (carboxylesterase, CarE), and A0A7J7HJ35 (cytochrome P450 family protein, CYP450) were obtained from the UniProt database. These proteins are critical metabolic enzymes in plants and play essential roles in pesticide metabolism and transport in plants. The small molecules were designated as ligands, and the proteins were designated as receptors for pairwise docking. Docking simulations were conducted using autodock vina. Scores were computed for protein–small-molecule docked complexes, which were subsequently analyzed and visualized using Pymol 1.1 and Discovery Studio 3.1.

### 2.9. Exposure Assessment and Risk Characterization

According to the food consumption and the quantity of pesticide residue identified in tea, the dietary exposure to pesticide residue was assessed. Pesticide residue levels surrounding maximum residue limits (MRLs) may trigger acute and chronic toxicity to consumers. According to FAO and WHO statistics, the average and highest daily tea intake in China was 0.0114 kg/standard person and 0.07588 kg/standard person, respectively [23]. The National Estimated Daily Intake (NEDI) and the National Estimated Short-Term Intake (NESTI) of pesticides for single doses and combinations were computed.

NEDI = F ∗ STMR/bw(3)

NESTI = LP ∗ HR/bw(4)

where F represents the average daily per capita consumption of tea, LP (large portion) represents the maximum consumption of tea, and bw denotes the body weight of consumers of 63 kg in China. The Supervised Trials Median Residue (STMR) was used to calculate NEDI, whereas the highest expected residue (HR) was used to calculate NESTI [24,25].

The risk quotient (RQ) method assessed the chronic and acute dietary risk of pesticide residues, defined as the ratio of dietary exposure to pesticides and acceptable daily intake (ADI) or acute reference dose (ARfD) values [24]. RQ > 1 demonstrated an unacceptable risk of residual pesticide for humans. In contrast, RQ < 1 demonstrated an acceptable risk to humans.

In light of the different degrees of degradation of pesticides throughout tea processing and brewing [26], dietary risk was further calculated in combination with processing factors to comprehend the dynamic changes in pesticides during green tea processing and brewing.

## 3. Results and Discussion

### 3.1. Method Validation

**Figure 1:**
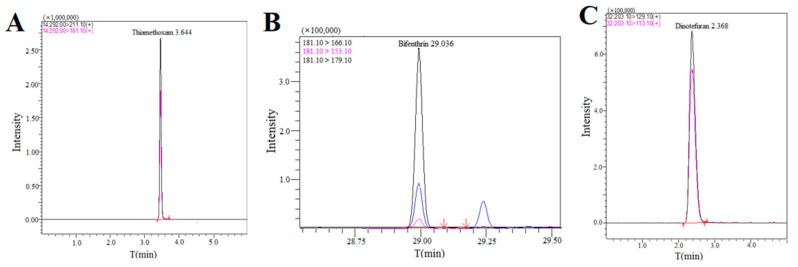
The chromatograms of thiamethoxam (**A**), bifenthrin (**B**), and dinotefuran (**C**) in tea.

The chromatograms of thiamethoxam, bifenthrin, and dinotefuran were illustrated in Figure 1. The validation results of each performance index of the method are shown in Table 1 (six replicates were performed). High linearity and correlation coefficient (r^2^ ≥ 0.9993) of the matrix-matched calibration curves were identified across five concentrations (1–250 μg/kg). The results indicated that the LOQs of five pesticides in tea were 1.00–2.50 μg/kg, alongside a recovery rate from 82.50% to 110.80%, while the RSDs were less than 9.32%. These findings exhibited satisfactory accuracy and precision of three pesticides within tea matrices.

### 3.2. Dissipation Kinetics of Individual and Joint Application in Tea

The dissipation curves of thiamethoxam, bifenthrin, and dinotefuran are depicted in Figure S1. The dissipation half-life, dissipation regressive equation, and R^2^ are shown in Table 2. The initial concentrations of thiamethoxam, bifenthrin, and dinotefuran in tea were 1.12, 2.95, and 7.88 mg/kg, with a half-life of 5.44, 9.81, and 10.16 days, respectively. The residual concentrations of active ingredients from pesticides in tea dissipated following first-order kinetics. The correlation coefficient of the degradation equation after individual or joint applications were 0.8032–0.8662, which showed appropriate agreement with first-order kinetics.

As outlined in Table S2, the residue of thiamethoxam rapidly decreased from the 3rd day (pre-harvest interval), reaching 75.69%. After the 14th day, the degradation attained over 96.08% and stabilized. For bifenthrin, the degradation rate reached 58.11% on the 3rd day and 77.92% on the 7th day (pre-harvest interval), and the concentration degradation rate reached more than 86.18% after the 14th day, remaining essentially stable. The degradation rate of dinotefuran reached 44.08% on the 3rd day, 60.77% on the 7th day (pre-harvest interval), and more than 83.84% on the 28th day. The degradation rate of dinotefuran slowed on the 14th day.

After the pre-harvest interval, the residual concentrations of dinotefuran in individual and in combination were 3.09 mg/kg and 2.27 mg/kg, respectively. These were lower than MRLs in China and Japan but significantly higher than MRLs in the EU (Table S3) [27,28,29]. The final residual amounts of other pesticides and binary combinations following the pre-harvest interval were lower than those of MRLs in China, Japan, and the EU.

In addition to the thiamethoxam + dinotefuran group, the concentration of the joint application group at the same time was higher compared with the individual pesticide spraying group in most cases, and it took a longer time to achieve the same degradation efficiency. For instance, the degradation concentration of thiamethoxam reached 0.27 mg/kg after three days of treatment, and it took seven days for the joint treatment of thiamethoxam + bifenthrin and thiamethoxam + dinotefuran to reach the same concentration. The degradation concentration of bifenthrin attained 0.65 mg/kg after seven days, and the degradation period of joint treatment with thiamethoxam + bifenthrin at the same concentration was extended to 7–14 days. Generally, the joint application of insecticides prolonged the degradation time of thiamethoxam and bifenthrin relative to individual pesticide application.

Previous individual investigations examined the dissipation behaviors of β-cyfluthrin and imidacloprid across different foods based on field trials, but the interaction of multiple pesticides on dissipation behavior following joint application has not been considered [30]. The joint application of various pesticides influences their respective degradation half-lives and ultimate residue concentrations [19]. Following the joint application of thiamethoxam and procymidone in tomatoes, the residual levels of these two pesticides were higher than the single application, suggesting that the interaction caused a slower dissipation rate of the two pesticides [31]. Bai et al. examined the dissipation dynamics and residual distribution of mixed dinotefuran and tolfenpyrad in tea throughout different regions of China. Notably, over 90% of dinotefuran in tea was degraded after 28 days following combined administration, consistent with our findings [32].

### 3.3. Comparison of Dissipation Behaviors between Individual and Joint Application

For thiamethoxam, when combined with bifenthrin or dinotefuran, the degradation rate slowed, and the degradation half-life correspondingly increased (5.58 and 5.97 days, respectively), with the final residual concentration increasing 1.41 and 1.64 times, respectively. This indicated an increased risk in the pre-harvest interval of thiamethoxam after the joint application. For bifenthrin, the half-life increased to 10.50 days when employed in combination with thiamethoxam, and the final residue increased 1.29 times. For dinotefuran, after being combined with thiamethoxam, the degradation rate was accelerated, and the half-life was reduced by 18.62%, suggesting that the combination reduced the pre-harvest interval of dinotefuran. Our results offered insights that interactions occurred in the dissipation dynamics of thiamethoxam, bifenthrin, and dinotefuran in tea, and that these interactions were dependent upon the specific pesticide.

### 3.4. Mechanism of Combined Dissipation Behaviors Following a Joint Application

The joint application of pesticides to control multiple pest species has attracted increased attention [33]. Few investigations have been performed on the interaction of various pesticides on their respective dissipation. The potential explanations for the variations in dissipation characteristics following joint application in tea were as follows.

Firstly, the variation in half-life between the different insecticides is linked to their chemical properties [25]. Bifenthrin possesses a high octanol/water partition coefficient (Kow), suggesting that it is likely to move through membranes and be readily absorbed and accumulated by plants [34]. Thiamethoxam is more easily broken down in the environment and has a shorter dissipative half-life due to its lower Kow and higher water solubility [35]. The metabolites of dinotefuran are more mobile and persistent, potentially leading to its long half-life [36].

Using the pkCSM platform (https://biosig.lab.uq.edu.au/pkcsm/, accessed on 30 November 2023), the pharmacokinetic and pharmacological properties of pesticides were predicted. The findings indicated that both thiamethoxam and bifenthrin can be substrates for P-glycoprotein, playing an important role in the absorption and transport of exogenous substances within plants [37]. When these two pesticides are sprayed in combination, competitive binding may occur, producing an extended half-life for their dissipation. Similarly, the predicted results indicated that the metabolite of dinotefuran, dinotefuran DN, is a substrate of P-glycoprotein. Due to its elevated water solubility, plants can more easily absorb and metabolize dinotefuran. Therefore, when thiamethoxam and dinotefuran were employed in combination, dinotefuran can be metabolized into dinotefuran DN, causing competitive transport metabolism with thiamethoxam, and the rate of thiamethoxam degradation decreased.

**Figure 2:**
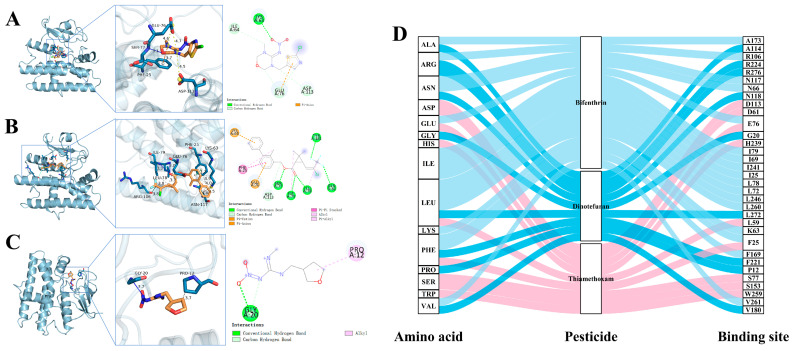
Molecular docking results of thiamethoxam (**A**), bifenthrin (**B**), and dinotefuran (**C**) with GST metabolizing proteins and interaction of amino acids and their binding sites (**D**).

Additionally, after absorbing exogenous substances, plants use diverse detoxifying defense mechanisms. CYP450, CarE, and GST are crucial metabolic enzymes in plants that play important roles in the metabolism and absorption of substances [38,39,40,41,42]. We employed molecular docking techniques to assess the interactions between three pesticides and metabolic enzymes, as depicted in Figures S2 and S3. The binding energies of thiamethoxam with GST, CarE, and CYP450 proteins were −5.3, −5.1, and −4.8 kcal/mol, respectively. For bifenthrin, the binding energies with GST, CarE, and CYP450 proteins were −8.7, −7.4, and −6.2 kcal/mol, respectively. Meanwhile, the binding energies of dinotefuran with GST, CarE, and CYP450 proteins were −5.8, −4.9, and −5.3 kcal/mol, respectively. Lower energy values suggested a higher degree of binding between the pesticide molecules and proteins. It was clear that all three pesticides have strong binding abilities with GST, suggesting its primary role in pesticide metabolism (Figure 2A–C). The competition between thiamethoxam and bifenthrin with GST enzymes may cause a decreased degradation rate following joint application.

Phenylalanine and glutamate at multiple sites participated in the binding of three pesticides and metabolic enzymes (Figure 2D and Figure S3), shown to be involved in plant immune defense [43,44]. Thiamethoxam bound to the 76th GLU amino acid residue of the receptor GST protein via two salt bridge interactions at distances of 4.6 Å and 4.7 Å, while bifenthrin also bound to the same residue via a hydrophobic interaction at a distance of 3.7 Å. Additionally, the 25th PHE amino acid residue was a binding target for thiamethoxam and bifenthrin, interacting with GST protein via π-cation interactions, hydrophobic forces, and π-π stacking forces. Therefore, when thiamethoxam and bifenthrin were simultaneously applied, competitive inhibition may occur, causing prolonged dissipation half-lives of these insecticides and altering their metabolism (Table 2).

Relative to thiamethoxam, dinotefuran exhibited an increased binding affinity of 9.43% and 10.42% toward GST and CYP450 proteins, respectively. While its binding affinity toward the CarE protein decreased by 3.92%, overall, the enhanced binding ability of dinotefuran to metabolic enzymes was identified in tea compared with thiamethoxam. Therefore, after the combined application of thiamethoxam and dinotefuran, non-competitive inhibition was found where dinotefuran exhibited stronger binding affinity, prioritizing its metabolism absorption over thiamethoxam, resulting in a shorter half-life of dinotefuran (Table 2).

In contrast, degradation by microorganisms is essential for pesticide dissipation [45,46,47]. Yu et al. determined that the presence of thiamethoxam and dinotefuran at low doses enhanced community diversity, while moderate and high levels of exposure caused a reduction in community diversity [25]. In our study, the initial residual amount of dinotefuran was higher than thiamethoxam, so the difference in concentration would present different impacts on the microbial community and lead to changes in dissipation behaviors following the joint application (Table S2), which is worthy of further study.

### 3.5. Risk Assessment for Individual and Joint Applications

Dissipation and pesticide residues following joint applications can pose a risk to human health. To examine long-term and short-term pesticide residue exposure, the average and highest residue levels of thiamethoxam, bifenthrin, and dinotefuran following individual and joint applications were employed (Table 3). Notably, these three pesticides undergo varying degrees of degradation throughout processing, impacting the risk of pesticide residues. Temperature, humidity, pH, moisture, and other environmental factors exert different degrees of influence on pesticide residues [48,49,50]. The degradation coefficients during green tea processing are 15.00% for thiamethoxam, 40.00% for bifenthrin, and 22.22% for dinotefuran [51,52]. In this study, we integrated degradation coefficients to model pesticide residue levels in green tea. Combined with dietary intake data, acute and chronic dietary risk assessment for green tea under single and combined pesticides applications was conducted.

The NEDIs of thiamethoxam, bifenthrin, and dinotefuran were 4.20 × 10^−5^, 7.08 × 10^−5^, and 4.35 × 10^−4^ mg kg^−1^ bw d^−1^, respectively. The NESTIs were 3.15 × 10^−4^, 5.09 × 10^−4^, and 3.04 × 10^−3^ mg kg^−1^ bw d^−1^, respectively. Chronic risk quotient (RQc) and acute risk quotient (RQa) were all below 0.71% and 5.09%, respectively, indicating that the chronic and acute dietary intake risks of the three pesticides were at acceptable levels when applied individually.

Under joint applications, when combined with bifenthrin or dinotefuran, the NEDI value of thiamethoxam was increased by 1.59 times and 1.89 times, and the NESTI value also increased by 1.44 times and 1.92 times, respectively. Similarly, under the binary combination of thiamethoxam and bifenthrin, both NEDI and NESTI values of bifenthrin were increased by about 1.4 times. Under the binary combination of thiamethoxam and dinotefuran, the NEDI and NESTI values of dinotefuran decreased to 0.73 and 0.79 times, respectively. Via risk assessment, the RQc in the joint applications was less than 1.04%, and the RQa was less than 7.33%, indicating that the chronic and acute dietary intake risks of consumers were acceptable following joint application.

In this study, the dynamic factors governing pesticide changes in the processing and brewing of green tea were outlined to offer a comprehensive assessment of the dietary risks associated with tea consumption. As the pesticide residues in fresh tea leaves harvested from field experiments were below the MRLs, and both acute and chronic toxicity risks were within acceptable levels, dietary risks were further reduced after degradation processes like processing and brewing (Table 3). However, no research has examined whether the coexistence of pesticides affects processing factors and transfer coefficients. Therefore, gaps should be filled for a complete understanding of the joint impact of the combined application on health effects through tea consumption.

Human dietary intake is made up of various foods, and pesticides may persist in different foods simultaneously. Neonicotinoids possess low molecular weight and high water solubility and can be transferred to grains, vegetables, and fruits. Therefore, multiple food sources can increase cumulative risk [53]. Pyrethroids tend to accumulate in biological tissues due to their lipophilicity, posing prolonged and chronic exposure risks to non-target organisms, and attracting increased attention to their toxicological effects [54] Therefore, the cumulative risk of the joint application of neonicotinoids and pyrethroids should be investigated in the future.

## 4. Conclusions

This study focused on dissipation behaviors and risk assessment of thiamethoxam, bifenthrin, and dinotefuran in tea following individual and joint application. Following combined application, dissipation half-lives and final concentrations were significantly influenced. Under combined administration of thiamethoxam and bifenthrin, half-lives were prolonged compared with their individual treatments, increasing to 5.58 and 10.50 days, respectively. When combined with dinotefuran, thiamethoxam’s half-life was extended to 5.97 days, and the residue following 28 days was 1.64 times higher than the individual application. Different pesticide combinations and application modes impacted residual concentration and dissipation behavior, potentially related to the interaction between pesticides and P-glycoproteins in the absorption and transport of exogenous substances within plants, as well as the metabolism of pesticides in tea, especially GST metabolism and binding to phenylalanine and glutamate residues. After the pre-harvest interval, whether administered alone or in combination, the pesticide residue concentrations were lower than China’s MRLs. However, given the multiple dietary sources and the accumulation of pesticides, the cumulative risks of pesticide combinations from various dietary sources cannot be overlooked.

## Figures and Tables

**Table 1:** The method validation of thiamethoxam, bifenthrin, and dinotefuran in tea matrix (n = 6).

Analyte	Retention Time (min)	Ion Pair (*m*/*z*)	Linear Regression Equation	(r2)	Linearity Range (μg/kg)	Recoveries (%)	RSD (%)	LOD (μg/kg)	LOQ (μg/kg)
Thiamethoxam	3.644	292.00 > 211.10 * 292.00 > 181.10	Y = 41,565.0X + 260,001	0.9993	1–200	82.5–90.3	5.14–6.72	0.3	1
Bifenthrin	29.036	181.10 > 166.10 * 181.10 > 153.10	Y = 1808.886X + 5330.985	0.9994	1–250	102.5–110.8	8.49–9.32	0.3	1
Dinotefuran	2.368	203.10 > 129.10 * 203.10 > 113.10	Y = 37,739.6X + 68,252.7	0.9993	2.5–200	86.3–102.2	6.81–7.58	0.8	2.5

* indicated quantitative ion pair.

**Table 2:** The dissipation regressive equation, correlation coefficient, and half-life of thiamethoxam, bifenthrin, and dinotefuran following individual or joint applications in tea.

Pesticides	Treatments	Dissipation Regressive Equation	R^2^	Rate Constant, k (Day^−1^)	Half-Life (Day)
Thiamethoxam	Thiamethoxam	C = 0.5787e^−0.1280t^	0.8318	0.13	5.44
	Thiamethoxam + Bifenthrin	C = 0.8101e^−0.1260t^	0.8662	0.13	5.58
	Thiamethoxam + Dinotefuran	C = 0.7603e^−0.1160t^	0.8596	0.12	5.97
Bifenthrin	Bifenthrin	C = 1.6691e^−0.0725t^	0.8032	0.07	9.81
	Thiamethoxam + Bifenthrin	C = 1.8384e^−0.0660t^	0.8531	0.07	10.50
Dinotefuran	Dinotefuran	C = 6.0540e^−0.0685t^	0.8459	0.07	10.16
	Thiamethoxam + Dinotefuran	C = 5.9530e^−0.0840t^	0.8603	0.08	8.27

**Table 3:** Exposure assessment and risk characterization of thiamethoxam, bifenthrin, and dinotefuran in tea.

Pesticides	Treatments	NEDI (×10^−5^, mg kg^−1^ bw d^−1^)	RQchronic (%)	NESTI (×10^−5^, mg kg^−1^ bw d^−1^)	RQacute (%)
Thiamethoxam	Thiamethoxam	4.20	0.05	31.53	0.03
	Thiamethoxam + Bifenthrin	6.67	0.08	45.46	0.05
	Thiamethoxam + Dinotefuran	7.92	0.10	60.61	0.06
Bifenthrin	Bifenthrin	7.08	0.71	50.88	5.09
	Thiamethoxam + Bifenthrin	10.38	1.04	73.28	7.33
Dinotefuran	Dinotefuran	43.49	0.22	303.72	0.30
	Thiamethoxam + Dinotefuran	31.88	0.16	240.57	0.24

## Data Availability

The original contributions presented in the study are included in the article/Supplementary Material, further inquiries can be directed to the corresponding author.

## Supplementary Materials

The following supporting information can be downloaded at: https://www.mdpi.com/article/10.3390/foods13193113/s1. Table S1: Weather conditions on the day of sampling. Table S2: Residue concentrations of thiamethoxam, bifenthrin, and dinotefuran after individual and joint applications in tea. Each treatment was performed in duplicate. Table S3: Maximum residue limits (MRLs) of thiamethoxam, bifenthrin, and dinotefuran in tea in China, Japan, and the European Union. Figure S1: Dissipation curves of thiamethoxam (A), bifenthrin (B), and dinotefuran (C) following individual or joint applications in tea. Each treatment was performed in triplicate, and bars represented standard error. Figure S2: Binding energy of three pesticides and three metabolic proteins (kcal/mol). Figure S3: Molecular docking results of thiamethoxam, bifenthrin, and dinotefuran with CarE (A–C) and CYP450 (D–F) metabolizing proteins, respectively.
