# Supplementary material for: Comparison of Combined Dissipation Behaviors and Dietary Risk Assessments of Thiamethoxam, Bifenthrin, Dinotefuran, and Their Mixtures in Tea
Source: Foods. 2024 Sep 29;13(19):3113. doi: 10.3390/foods13193113 (PMC11475861; doi:10.3390/foods13193113)
Supplement: Supplementary file 1 [file foods-13-03113-s001.zip › foods-3223849-supplementary.pdf]

Supplementary material

Catalogue

**Table S1**	Weather conditions on the day of sampling
**Table S2**	Residue concentrations of thiamethoxam, bifenthrin, and dinotefuran after individual and joint applications in tea. Each treatment was performed in duplicate.
**Table S3**	Maximum residue limits (MRLs) of thiamethoxam, bifenthrin and dinotefuran in tea in China, Japan and the European Union
**Figure S1**	Dissipation curves of thiamethoxam (A), bifenthrin (B), and dinotefuran (C) following individual or joint applications in tea. Each treatment was performed in triplicate, and bars represented standard error.
**Figure S2**	Binding energy of three pesticides and three metabolic proteins (kcal/mol).
**Figure S3**	Molecular docking results of thiamethoxam, bifenthrin and dinotefuran with CarE (A-C) and CYP450 (D-F) metabolizing proteins, respectively.

Table S1. Weather conditions on the days of sampling

Sampling date	Interval days (day)	Weather conditions (Temperature, humidity, weather)
9.22	0.08	20-28℃, 90%, cloudy
9.23	1	17-27℃, 56%, cloudy
9.25	3	19-24℃, 83%, cloudy to light rain
9.27	5	20-26℃, 89%, cloudy to light rain
9.29	7	20-27℃, 94%, cloudy
10.6	14	15-18℃, 85%, overcast
10.13	21	12~21°C, 90%, cloudy
10.20	28	14~20°C, 70%, cloudy

Table S2. Residue concentrations of thiamethoxam, bifenthrin, and dinotefuran after individual and joint applications in tea. Each treatment was performed in duplicate.

Pesticides	Treatments		Concentrations (mg/kg)									
			0.08	1	3	5	7	14	21	28	35	60
Thiamethoxam	Thiamethoxam	1	1.35	1.04	0.31	0.29	0.18	0.07	0.05	0.04	0.03	0.02
		2	0.9	0.68	0.24	0.27	0.15	0.02	0.03	0.02	0.02	0.03
		Mean	1.12	0.86	0.27	0.28	0.16	0.04	0.04	0.03	0.03	0.03
	Thiamethoxam+ Bifenthrin	1	1.6	1.34	0.44	0.48	0.32	0.06	0.09	0.07	0.04	0.02
		2	0.98	1.04	0.42	0.32	0.18	0.06	0.04	0.02	0.03	0.02
		Mean	1.29	1.19	0.43	0.4	0.25	0.06	0.06	0.05	0.03	0.02
	Thiamethoxam+ Dinotefuran	1	1.21	1.14	0.59	0.56	0.26	0.06	0.07	0.07	0.06	0.02
		2	0.91	0.88	0.44	0.36	0.2	0.07	0.05	0.04	0.03	0.02
		Mean	1.06	1.01	0.52	0.46	0.23	0.06	0.06	0.05	0.05	0.02
Bifenthrin	Bifenthrin	1	3.17	2.2	1.21	0.93	0.7	0.39	0.4	0.21	0.31	0.07
		2	2.74	1.49	1.27	0.92	0.6	0.43	0.45	0.36	0.2	0.1
		Mean	2.95	1.85	1.24	0.92	0.65	0.41	0.43	0.29	0.26	0.08
	Thiamethoxam+ Bifenthrin	1	2.61	2.49	1.4	1.04	1.01	0.53	0.59	0.36	0.3	0.18
		2	2.4	2.22	1.38	0.98	0.9	0.48	0.4	0.37	0.35	0.25
		Mean	2.51	2.35	1.39	1.01	0.96	0.5	0.5	0.37	0.32	0.22
Dinotefuran	Dinotefuran	1	7.83	7.52	4.75	4.67	3.24	1.57	1.53	1.45	0.87	0.12
		2	7.92	7.41	4.06	3.85	2.94	1.16	1.24	1.1	0.75	0.11
		Mean	7.88	7.47	4.4	4.26	3.09	1.37	1.38	1.27	0.81	0.12
	Thiamethoxam+ Dinotefuran	1	8.97	7.01	4.48	3.93	2.57	1.02	1.12	0.69	0.82	0.1
		2	9.25	6.82	4.26	3.21	1.96	1.12	1.07	0.85	0.52	0.13
		Mean	9.11	6.92	4.37	3.57	2.27	1.07	1.1	0.77	0.67	0.11

Table S3. Maximum residue limits (MRLs) of thiamethoxam, bifenthrin and dinotefuran in tea in China, Japan and the European Union (mg/kg).

Pesticide	China	Japan	European Union
Thiamethoxam	10	20	20
Bifenthrin	5	30	30
Dinotefuran	20	25	0.01

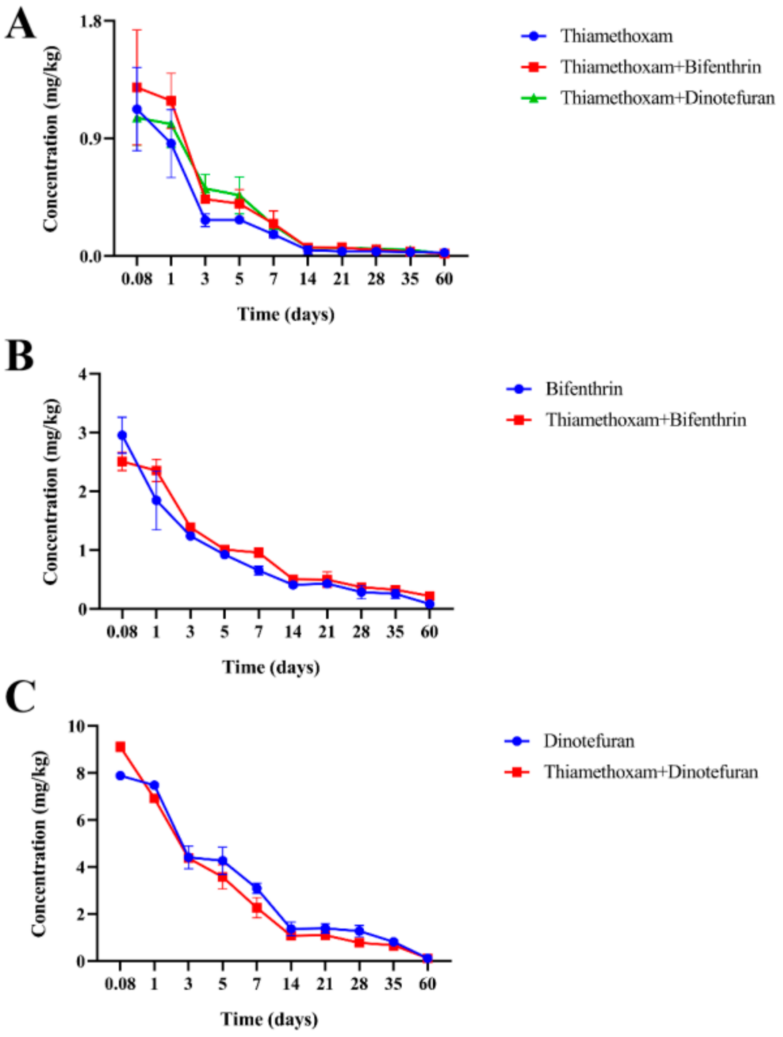

Figure S1 Dissipation curves of thiamethoxam (A), bifenthrin (B), and dinotefuran (C) following individual or joint applications in tea. Each treatment was performed in triplicate, and bars represent standard error.

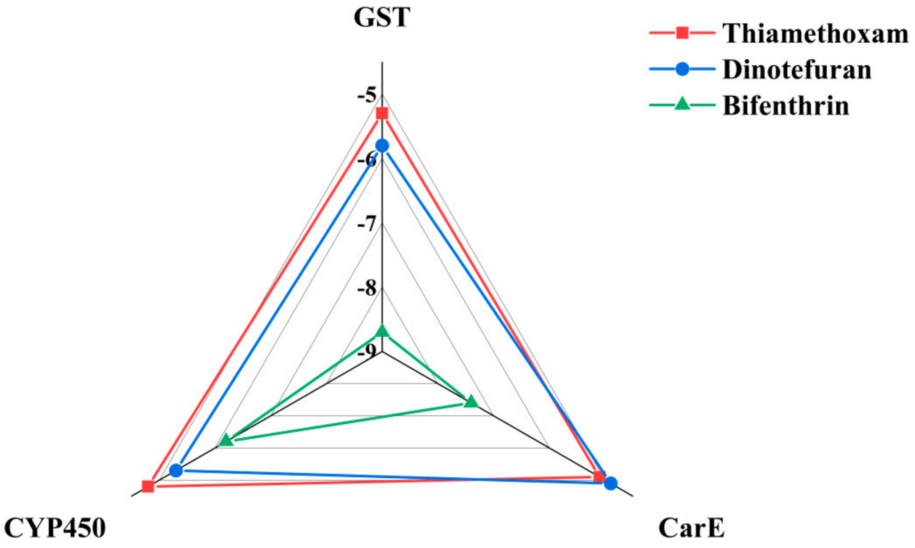

Figure S2. Binding energy of three pesticides and three metabolic proteins (kcal/mol).

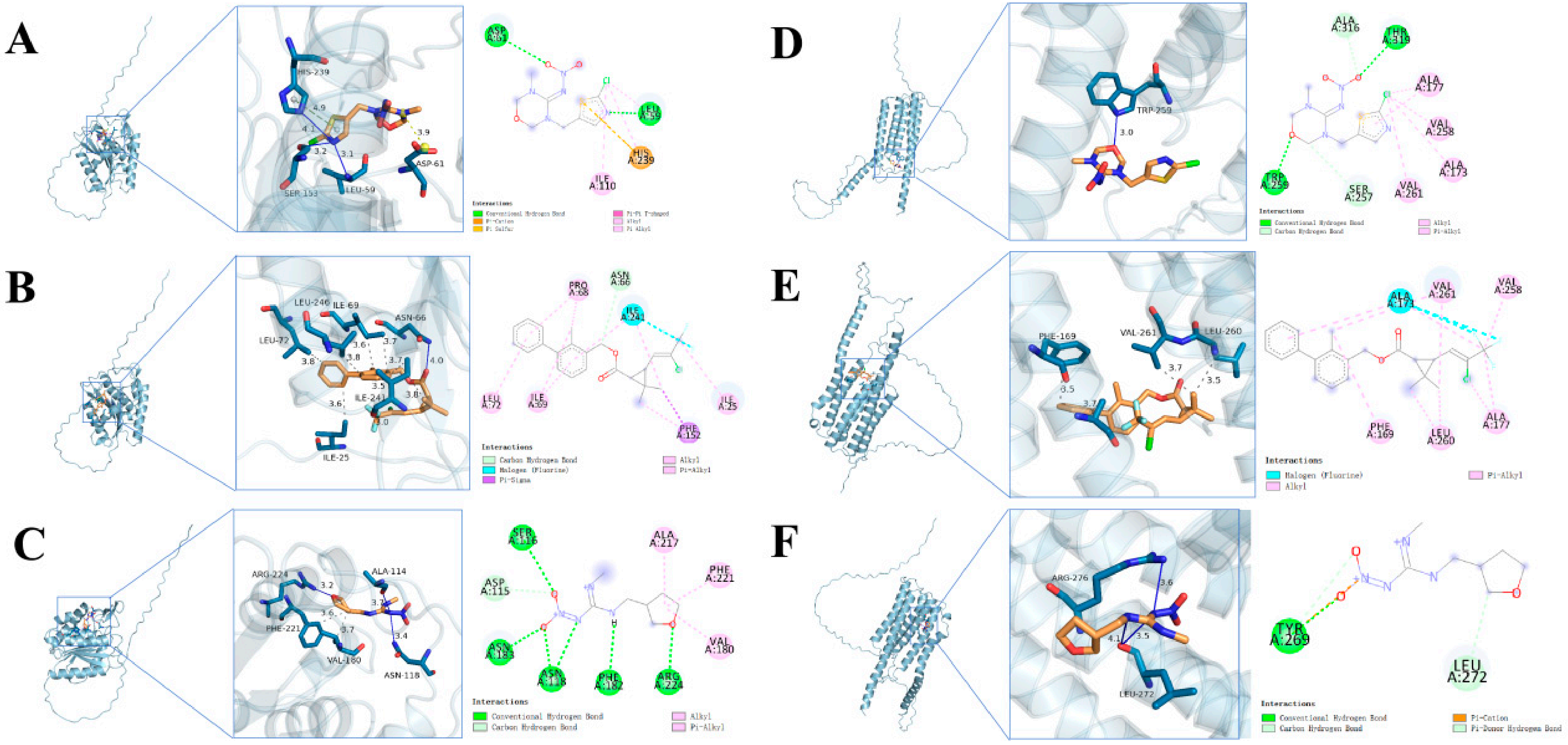

Figure S3. Molecular docking results of thiamethoxam, bifenthrin and dinotefuran with CarE (A-C) and CYP450 (D-F) metabolizing proteins, respectively.
